# *In vitro* and *in vivo* studies on the effect of a mitochondrial fusion promoter on Leydig cell integrity and function

**DOI:** 10.3389/ftox.2024.1357857

**Published:** 2024-03-06

**Authors:** Samuel Garza, Chantal Sottas, Hovhannes J. Gukasyan, Vassilios Papadopoulos

**Affiliations:** Department of Pharmacology and Pharmaceutical Sciences, Alfred E. Mann School of Pharmacy and Pharmaceutical Sciences, University of Southern California, Los Angeles, CA, United States

**Keywords:** testosterone, steroidogenesis, hypogonadism, bioenergetics, mitochondrial fusion, aging, toxicity

## Abstract

**Background:** The interstitial testicular Leydig cells are responsible for the production of testosterone, which functionally deteriorate with normal aging. Decreased expression of mitochondrial steroidogenic interactome proteins and diminished mitochondrial function in aging Leydig cells suggest that mitochondrial dynamics play a role in maintaining adequate levels of testosterone. Optic atrophy 1 (OPA1) protein regulates mitochondrial dynamics and cristae formation in many cell types. Previous studies showed that increasing OPA1 expression in dysfunctional Leydig cells restored mitochondrial function and recovered androgen production to levels found in healthy Leydig cells. These findings suggested that mitochondrial dynamics may be a promising target to ameliorate diminished testosterone levels in aging males.

**Methods:** We used twelve-month-old rats to explore the relationship between mitochondrial dynamics and Leydig cell function. Isolated Leydig cells from aged rats were treated *ex vivo* with the cell-permeable mitochondrial fusion promoter 4-Chloro-2-(1-(2-(2,4,6-trichlorophenyl)hydrazono)ethyl) phenol (mitochondrial fusion promoter M1), which enhances mitochondrial tubular network formation. In parallel, rats were treated with 2 mg/kg/day M1 for 6 weeks before Leydig cells were isolated.

**Results:** *Ex vivo* M1-treated cells showed enhanced mitochondrial tubular network formation by transmission electron microscopy, enhanced Leydig cell mitochondrial integrity, improved mitochondrial function, and higher testosterone biosynthesis compared to controls. However, *in vivo* treatment of aged rats with M1 not only failed to re-establish testosterone levels to that of young rats, it also led to further reduction of testosterone levels and increased apoptosis, suggesting M1 toxicity in the testis. The *in vivo* M1 toxicity seemed to be tissue-specific, however.

**Conclusion:** Promoting mitochondrial fusion may be one approach to enhancing cell health and wellbeing with aging, but more investigations are warranted. Our findings suggest that fusion promoters could potentially enhance the productivity of aged Leydig cells when carefully regulated.

## 1 Introduction

The interstitial testicular Leydig cells, which are responsible for the production of circulating testosterone levels, functionally deteriorate with normal aging (Midzak et al., 2009; Beattie et al., 2015; Zirkin and Papadopoulos, 2018). Although numerous studies have deepened our understanding of the deterioration of Leydig cell health with aging (Papadopoulos and Zirkin, 2021), the specific changes that result in mechanistic decline are not fully understood. Numerous changes in the cellular environment have been associated with a decline in steroidogenesis, including reductions in the expression of steroidogenic enzymes (Culty et al., 2002; Rone et al., 2012), imbalanced antioxidant levels (Beattie et al., 2015), reactive oxygen species production (Sokanovic et al., 2021), and reduced mitochondrial function (Midzak et al., 2009; Garza et al., 2022). Mitochondrial dynamics play a central role in maintaining cellular integrity and meeting ever-changing cellular demands (Friedman and Nunnari, 2014). The capacity of the mitochondria to balance bioenergetics and cellular stressors is mediated in part through mitochondrial dynamics, which can alter the cellular environment (Friedman and Nunnari, 2014). Understanding the interplay between aging and changes in mitochondrial function will lead to a better understanding of the role of mitochondrial dynamics in the age-related functional decline of Leydig cells.

Leydig cell steroidogenesis is orchestrated by a sequential series of signaling and metabolic steps that are highly regulated (Tremblay, 2015). Central to this process is a protein scaffold known as the steroidogenic interactome (SITE), which contains numerous steroidogenic proteins (Rone et al., 2009; Rone et al., 2012). This critical complex of proteins plays a pivotal role in the translocation of cholesterol from the cytosol into the mitochondria for steroidogenesis (Liu et al., 2006; Midzak et al., 2011). Numerous SITE proteins participate in the transfer of cholesterol, the precursor to all steroid hormones (Li et al., 2018; Garza et al., 2022). The expression of the proteins in SITE is highly regulated by multiple signaling pathways and transcription factors (LaVoie and King, 2009; Fan and Papadopoulos, 2021; de Mattos and VigerTremblay, 2022). However, with aging, these pathways can become compromised (Papadopoulos and Zirkin, 2021; Sokanovic et al., 2021; Garza et al., 2022), leading to insufficient testosterone production and potentially contributing to the physiological changes observed in older males (Zirkin and Chen, 2000; Tajar et al., 2010; Bhasin and Basaria, 2011; Fraietta et al., 2013). Importantly, the expression of SITE proteins is reduced in aged Leydig cells (Zirkin and Chen, 2000; Culty et al., 2002), suggesting that compromised protein-protein interactions are likely responsible for reduced cholesterol delivery and testosterone production (Rone et al., 2009; Rone et al., 2012). Although the regulation of Leydig cell steroidogenesis is well studied, the development and progression of Leydig cell dysfunction with aging is not yet fully understood.

Mitochondria are highly dynamic organelles responsible for orchestrating numerous essential cellular functions (Kasahara and Scorrano, 2014). They regulate bioenergetics with inherent adaptive mechanisms that enable them to respond to cellular demands (Frezza et al., 2006; Patten et al., 2014; Cogliati et al., 2016). Mitochondrial dynamics, including both fusion and fission, are involved in the regulation of cristae formation (Cogliati et al., 2013; Cogliati et al., 2016), not only altering the formation of the cristae and the mitochondrial network, but also influencing various other cellular processes (Frezza et al., 2006; Gomes et al., 2011; Cogliati et al., 2013; Friedman and Nunnari, 2014; Patten et al., 2014; Cogliati et al., 2016; Giacomello et al., 2020), further underscoring the versatility of these organelles. Mitochondrial membrane fusion is facilitated by specific mitochondrial dynamic proteins, of which the optic atrophy 1 (OPA1) protein is especially crucial (Patten et al., 2014; Del Dotto et al., 2018). OPA1 aids in the merging of the inner and outer mitochondrial membranes, supporting the formation of specialized regions known as contact sites (Song et al., 2007; Mishra et al., 2014; Ding et al., 2020). These sites act as conduits, streamlining the import of essential cellular cargo into the mitochondria (Schleyer and Neupert, 1985; Mishra et al., 2014).

It has been hypothesized that contact sites play a pivotal role in the translocation of cholesterol into the mitochondria (Thomson, 1998; Thomson, 2003). Given that cholesterol transport into the mitochondria is a crucial step in the synthesis of steroid hormones (Duarte et al., 2012), the interplay between mitochondrial dynamics and steroidogenesis may be essential to the maintenance of steroidogenic function. Data suggest that the regulation of mitochondrial contact sites may be involved in the expression of key steroidogenic proteins (Garza et al., 2022; Garza et al., 2023). Mitochondrial fusion, which is responsible for the formation and maintenance of contact sites (Brdiczka et al., 1998), deteriorates with aging (Jang et al., 2018), suggesting that age-related T decline may be a result of declining contact site integrity (Thomson, 2003; Rone et al., 2012). Fusion has been shown to be an integral requirement for steroidogenesis, and its inhibition diminishes steroid formation (Duarte et al., 2012). In our previous studies it was shown that enhancing mitochondrial fusion in dysfunctional Leydig cells produces functional gains and improvements in mitochondrial function (Garza et al., 2022).

Herein, we have investigated the role of mitochondrial dynamics, particularly fusion, in the regulation of Leydig cell steroidogenic function using a mitochondrial fusion promoter *in vivo* to shed light on how changes in mitochondrial dynamics can impact the overall steroidogenic capacity of aged Leydig cells and thereby influence the broader health of aging males. Previous studies indicated a beneficial effect for increased mitochondrial fusion on Leydig cell function in cell models (Garza et al., 2022), and our current study confirmed these data and extended them by examining the effect *in vivo*. Our results show that the mitochondrial fusion promoter M1, when injected daily, resulted in decreased testosterone formation, steroidogenic protein levels, and cell health, suggesting Leydig cell toxicity. These contradictory results suggest that mitochondrial fusion may help steroidogenic function but under intermittent isolated conditions. Additional research on the balance between mitochondrial fusion and steroidogenic function may enhance our understanding of Leydig cell deterioration.

## 2 Materials and methods

### 2.1 Animals

Sprague-Dawley rats aged 9 months to 1 year were maintained according to protocols approved by the Institutional Animal Care and Use Committee of the University of Southern California (Protocol #20791). Mitochondrial fusion promoter M1 (4-Chloro-2-(1-(2- (2,4,6-trichlorophenyl) hydrazono)ethyl)phenol, Sigma-Aldrich) (Wang et al., 2012) was dissolved in DMSO and diluted to 2 mg/kg/day for each animal. Animals were separated into three groups (n = 4 per group): untreated (control), DMSO-only control, or 2 mg/kg/day M1 (M1) daily for 6 weeks. The concentration of M1 and treatment duration used was chosen based on previous studies (Ding et al., 2020). Rats were sedated under isoflurane during tail blood collection throughout the study. Briefly, anesthesia was induced with isoflurane in an induction chamber. The animal was placed in the induction chamber and the isoflurane levels were turned to 2%–3% with a flow rate of 0.8–1.0 L/min. Once the animal was under, the gas supply to the induction chamber was turned off, the animal was removed from the induction chamber and mounted with a nose cone. The gas flow was restored at levels of 1.5%–2% at a flow rate of 0.4–0.8 L/min while tail blood was collected. All rats were euthanized by CO2 inhalant followed by decapitation by guillotine. Rats were placed into the CO2 chamber with flow rate between 30% and 50%. Thereafter, the rat was decapitated by guillotine, trunk blood was collected, and plasma was separated by centrifugation at 2000 *g* for 15 min, stored at −80°C, and used for determination of circulating testosterone levels.

### 2.2 Purification of Leydig cells with magnetic activated cell sorting (MACS)

Leydig cells were extracted using magnet-activated cell sorting (MACS), as previously described (Ji et al., 2022). Briefly, testes were collected from rats after euthanasia, washed and placed in ice-cold PBS. Testes were then decapsulated, and the testicular milieu was digested in 1 mg/mL collagenase in DMEM/F12 medium containing 0.1% bovine serum albumin (BSA) for 30 min at 34°C in a slow shaking incubator at 90 cycles/min. The digested testicular milieu was then filtered through a 70 µM pore nylon mesh. Cells were pelleted, then washed with DMEM/F12 medium, and resuspended in Ca^2+^, Mg^2+^-free Hank’s balanced salt solution containing 0.1% BSA before sorting.

Briefly, following the manufacturer’s protocol, testicular cell pellets were suspended in ice-cold IMag buffer at 2 × 10^7^ cells/mL with prolactin receptor (PRLR) antibody (1:150) for 30 min at 4°C. The labeled cell suspension was washed twice with IMag buffer and labeled with anti-mouse IgG1 magnetic beads (1:20) at 4°C for 30 min. The labelled cell suspension was placed into the IMag Cell Separation Magnet holder (BD Biosciences, US) for 8 min. The supernatant was then removed, and the positive fraction was resuspended in IMag buffer and placed in the magnet holder for an additional 4 min. This resuspension was performed two more times to purify the cell population. Purification was verified by both staining for 3β-hydroxysteroid dehydrogenase and flow cytometry.

The purity of Leydig cells was determined by a 3β-hydroxysteroid dehydrogenase chemical reaction as described previously (Browning et al., 1981). In brief, Leydig cell fractions were incubated for 30 min at 37°C with the substrate dehydroepiandrosterone (100 g/mL; Sigma-Aldrich, St. Louis, MO, United States) in 0.07 M phosphate buffer (pH 7.2) containing 1 mg/mL nicotinamide, 6 mg/ml g-NAD, and 1.5 mg/mL nitro blue tetrazolium (Sigma-Aldrich). In parallel, flow cytometry was performed after the fractions were incubated with a PE-conjugated goat anti-mouse IgG fluorescent secondary antibody in darkness for 1 h. PRLR + cells were analyzed using a Fortessa X20 Flow Cytometer (BD Biosciences).

### 2.3 Immunoblot analysis

Proteins were extracted using RIPA buffer supplemented with protease inhibitors. Protein concentrations were measured using a Pierce BCA Protein Assay Kit (ThermoFisher Scientific, Waltham, MA, United States). Sodium dodecyl sulfate-polyacrylamide gel electrophoresis was performed using 1 μg/μL of purified protein on a 4%–20% Tris-glycine gradient gel (Bio-Rad, Hercules, CA, United States). Protein bands were electro-transferred onto a polyvinylidene fluoride membrane and blocked with 5% BSA for 30 min. Anti-OPA1 mouse monoclonal antibody clone 1E8-1D9 that reacts with mouse, rat, and human protein was from ThermoFisher Scientific (UniProt ID: O60313). Rabbit monoclonal antibody against cleaved Caspase-3 (UniProt ID: P42574) from Cell Signaling Technology was used to visualize the protein. TSPO protein expression was assessed using an affinity purified rabbit anti-peptide antibody raised against the mouse TSPO C-terminal sequence as previously described (Payne and Hardy, 2007; Batarseh and Papadopoulos, 2010). This antiserum detects human, mouse, and rat TSPO. Other antibodies against CYP11A1 (UniProt ID: P05108), STAR (UniProt ID: P49675), VDAC1 (UniProt ID: P21796), RIPK1 (UniProt ID: T1503), and MFN2 (UniProt ID: O95140) were used as appropriate. Membranes were incubated with primary antibody overnight at 4°C at 1:1,000 dilution and secondary antibody at 1:5,000 for 1 h at room temperature. Membranes were then quenched with Radiance Peroxide and Radiance Plus (Azure Biosystems, Dublin, CA, United States) and subsequently imaged with an Azure c600 system (Azure Biosystems). Membranes were then stripped with Restore Western blot Stripping Buffer (ThermoFisher Scientific) and incubated with an antibody against the housekeeping protein GAPDH (1:1,000) (Proteintech, Rosemont, IL, United States) for normalization used as a control. Protein expression levels were measured using the ImageJ software, in which the density of the protein band of interest was compared with the GAPDH level for that particular band.

### 2.4 Measurement of testosterone

Cells were treated with 50 ng/mL human chorionic gonadotropin (hCG; National Hormone and Peptide Program, Harbor-UCLA Medical Center Torrance, CA, United States) or control media and incubated for various time points at 37°*C. Media* were collected for testosterone measurement using the Testosterone ELISA Kit (Cayman Chemical, Ann Arbor, MI, United States). Similarly, blood serum was collected and evaluated using the same Testosterone ELISA Kit.

### 2.5 Measurement of cellular respiratory function

Cultured cells (1 × 10^4^) and isolated primary Leydig cells (3 × 10^4^) were plated onto Seahorse XF Cell Culture Microplates overnight (Agilent Technologies, Santa Clara, CA, United States). Cell media were replaced with Agilent Seahorse XF DMEM Medium supplemented with 1 mM glucose, 1 mM pyruvate, and 2 mM glutamine and incubated at 37°C in a non-CO_2_ incubator. Cells were evaluated using a Seahorse XF Cell Mito Stress Test Kit or a Seahorse XF Real-Time ATP Rate Assay Kit according to the manufacturer’s specifications. Briefly, working solutions of oligomycin (2.5 µM), FCCP (2.0 µM) and Rot/AA (0.5 µM) were prepared and loaded into a sensor cartridge that had been hydrated in Seahorse XF Calibrant at 37°C in a non-CO_2_ incubator overnight. The assay was performed using a Seahorse XFe96 Analyzer with templates designed in Wave 2.6.1.

### 2.6 Transmission electron microscopy (TEM)

Cells were imaged by TEM at the USC Core Center of Excellence in Nano Imaging. Cells were primarily fixed using 2.5% glutaraldehyde, 2% paraformaldehyde, 0.1 M HEPES, and 0.115 M Sucrose. After washing with 0.1 M cacodylate, cells were placed in a secondary fixative containing 1% osmium tetroxide. Cells were then stained with uranyl acetate and dehydrated with a series of 30%–100% EtOH washes. The dehydrated cells were transitioned to a microfuge tube using propylene oxide and infiltrated using increasing concentrations of Poly/Bed 812 epoxy resin. The cell-containing-block was sectioned using a Leica EM UC6 Ultramicrotome (Leica Biosystems, Nussloch, Germany) and examined on a FEI Talos F200C G2 Biological Transmission Electron Microscope (ThermoFisher Scientific). The images produced were quantified using ImageJ software, where the number of observable mitochondria and their size were measured to quantify mitochondrial biogenesis and mitochondrial size.

### 2.7 Predictions of ADME properties

The Simplified Molecular Input Line Entry System format structure of M1 was obtained from the PubChem database and uploaded into the GastroPlus software 9.8 version (Simulations Plus Inc., United States). The Absorption, Distribution, Metabolism, Excretion and Toxicity (ADMET) Predictor software 11 version and PKPlus modules of GastroPlus v9.8 were used to simulate physicochemical and pharmacokinetic properties, respectively, in normal rats. Formulation and dosing parameters for the model were defined as following: 2 mg/kg/day injected into the rat model over 6 weeks (i.e., subsequent dosing of 2 mg/kg at an interval of every 24 h for the duration of 1,008 h, to mimic *in vivo* study conditions).

### 2.8 Statistical analysis

Data from experiments performed in technical triplicate are expressed as the mean ± standard error of the mean. GraphPad Prism (v.7; GraphPad Software, San Diego, CA, United States) was used for graphical presentation and statistical analysis. Analysis was performed using a Student’s t-test or ANOVA with multiple comparisons where appropriate. Results were considered statistically significant at *p* < 0.05.

## 3 Results

### 3.1 Characterization of MACS-isolated Leydig cells

**FIGURE 1:**
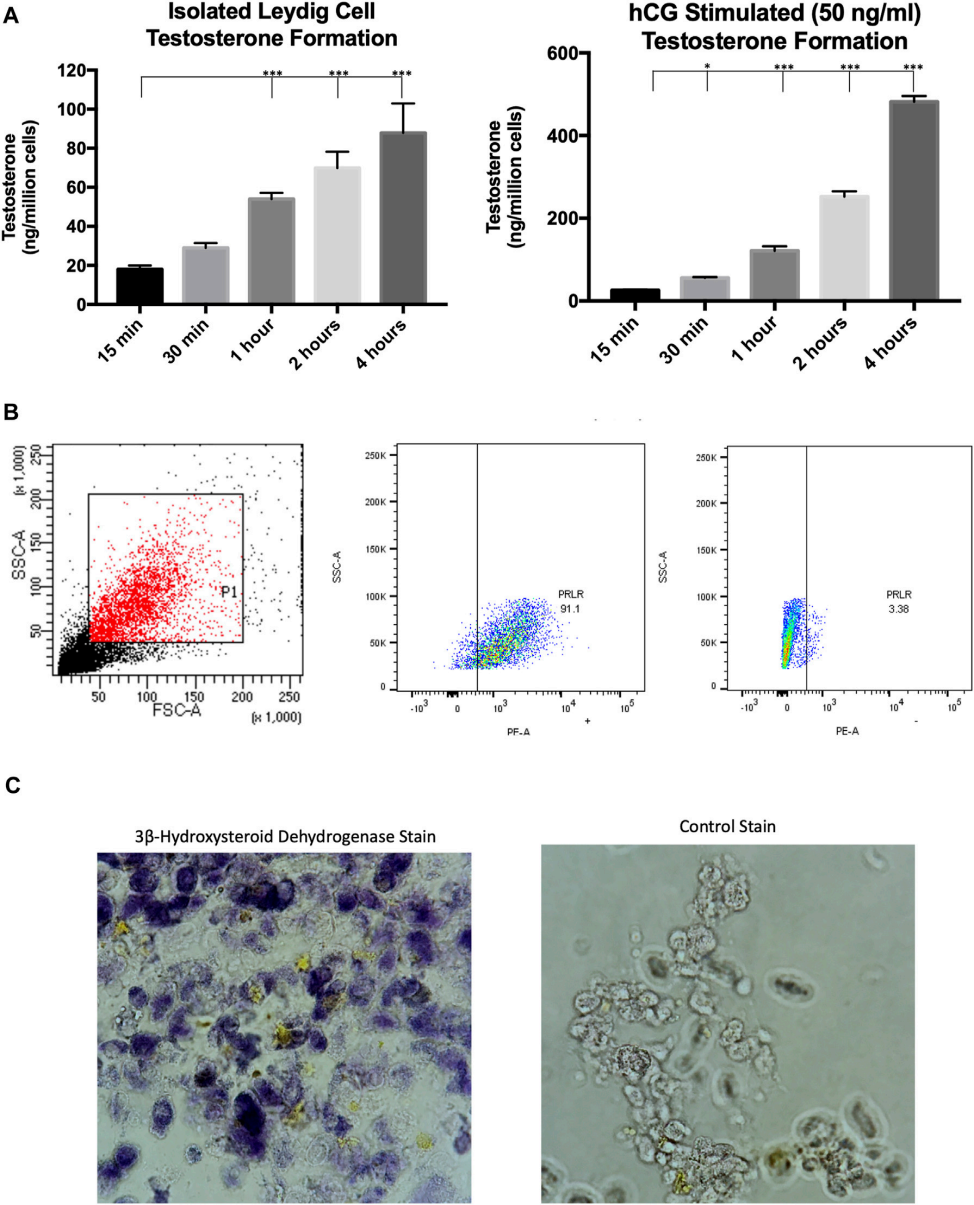
Characterization of MACS-isolated Leydig cells. **(A)** Time course of testosterone formation levels of MACS-isolated cells under basal and hormone-stimulated (hCG) conditions. **(B)** Gating strategy and flow cytometry analysis for PRLR + cells. **(C)** Staining for 3β-hydroxysteroid dehydrogenase enzymatic activity. Data are presented as mean ± SEM. *p < .05, **p. < .01, and ***p < .001 by ANOVA.

Our previous studies isolated primary Leydig cells using isosmotic continuous Percoll (Gibco Inc.) gradients generated by centrifugation and subsequent BSA gradients (Browning et al., 1981). It was recently demonstrated that Leydig cells could be isolated from adult rats using magnetic-activated cell sorting (Ji et al., 2022), saving a considerable amount of time in experimental procedures. Evidence indicates that the prolactin receptor is a unique Leydig cell surface marker (Green et al., 2018; Guan et al., 2022), which was targeted in our MACS protocol. This cell sorting technique was used to obtain a purified Leydig cell population that produced testosterone under both basal and hormone stimulated conditions (Figure 1A). To further confirm that the sorted cell population was Leydig cells, the recovered cells were incubated with a PE-conjugated antibody and subsequent flow cytometry analysis revealed >90% of isolated cells expressed PRLR (Figure 1B). Additionally, the Leydig cell population was reaffirmed after staining for 3β-hydroxysteroid dehydrogenase activity (Figure 1C), an enzyme specific to steroidogenic cells.

### 3.2 Promoting mitochondrial fusion in Leydig cells from aged rats enhances bioenergetics

**FIGURE 2:**
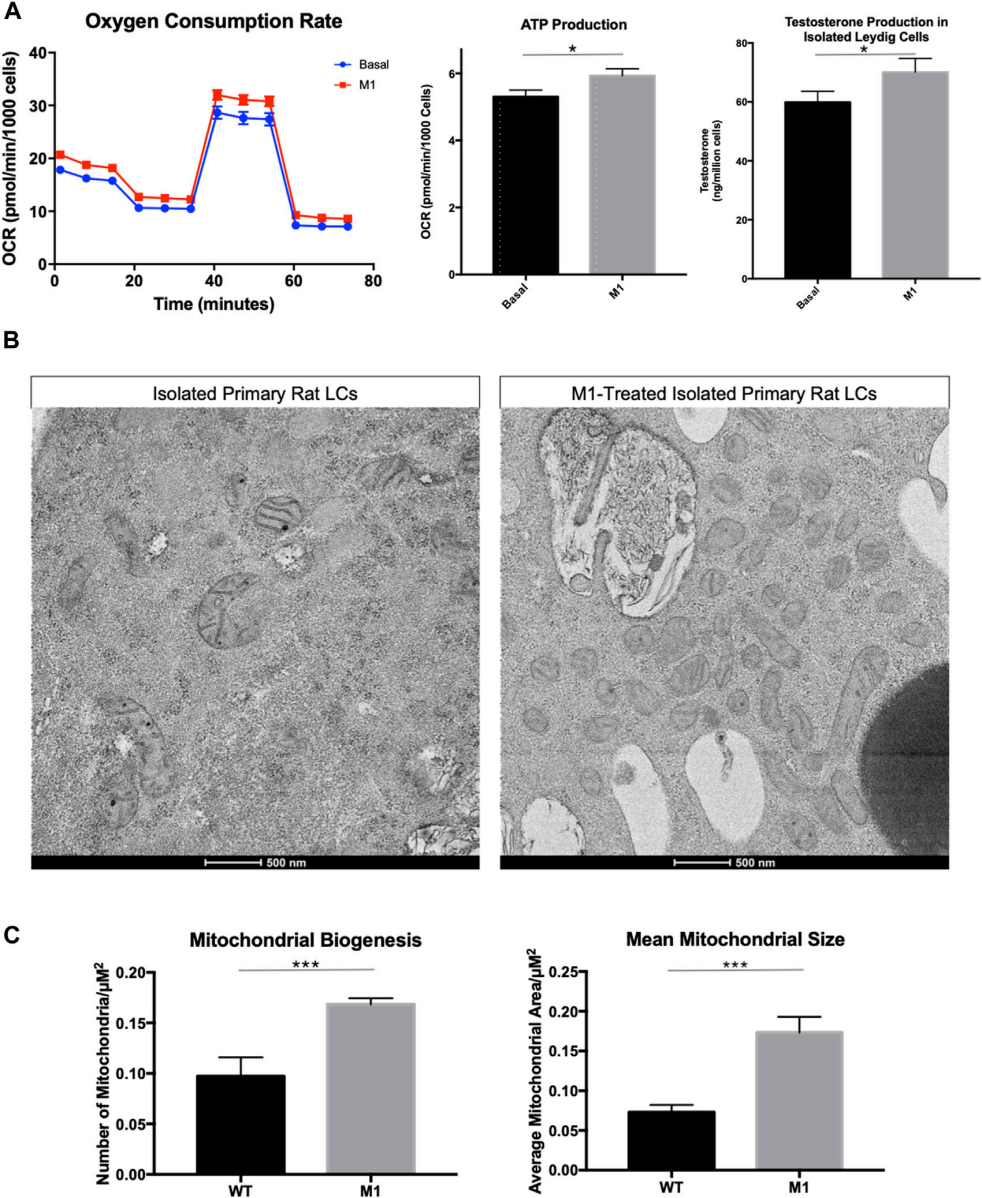
Promoting mitochondrial fusion in Leydig cells isolated from aged rats enhances bioenergetics. **(A)** Oxygen consumption, ATP production, and steroid hormone production of MACS-isolated Leydig cells from M1-treated and control (basal) aged rats. **(B)** TEM imaging of MACS-isolated Leydig cells from treated and untreated aged rats highlighting mitochondrial morphology. **(C)** Characteristics of mitochondrial health, mitochondrial biogenesis and mitochondrial size for mitochondria from MACS-isolated Leydig cells from M1-treated and control (wild-type) aged rats. Data are presented as mean ± SEM (n = 4). *p < .05, **p. < .01, and ***p < .001 by Student’s t-test. Scale bar, 500 nm. TEM, transmission electron microscopy.

To investigate the relationship between mitochondrial fusion and function in aged Leydig cells, we induced mitochondrial fusion *ex vivo* by treating isolated Leydig cells with cell-permeable M1, which has been shown to promote mitochondrial tubular network formation, increase OPA1 expression, and increase ATP levels (Wang et al., 2012; Ding et al., 2020). Treatment with M1 resulted in increased mitochondrial function and increased steroid biosynthesis (Figure 2A). The oxygen consumption rate and hormone production of the M1 treated cells showed improved cellular function. Based on our previous findings (Garza et al., 2022), we had expected that mitochondrial structure would improve after M1 treatment. Dysfunctional mitochondria accumulate with aging and present abnormal shape and structure. Using TEM to visualize mitochondrial morphology, we found that untreated aged Leydig cells had fewer healthy mitochondria when compared to the M1-treated Leydig cells (Figure 2B). The treated aged Leydig cells also contained more mitochondria with normal cristae structure (Figure 2C).

### 3.3 Administration of M1 leads to decreased in weight and testosterone levels in aged rats

**FIGURE 3:**
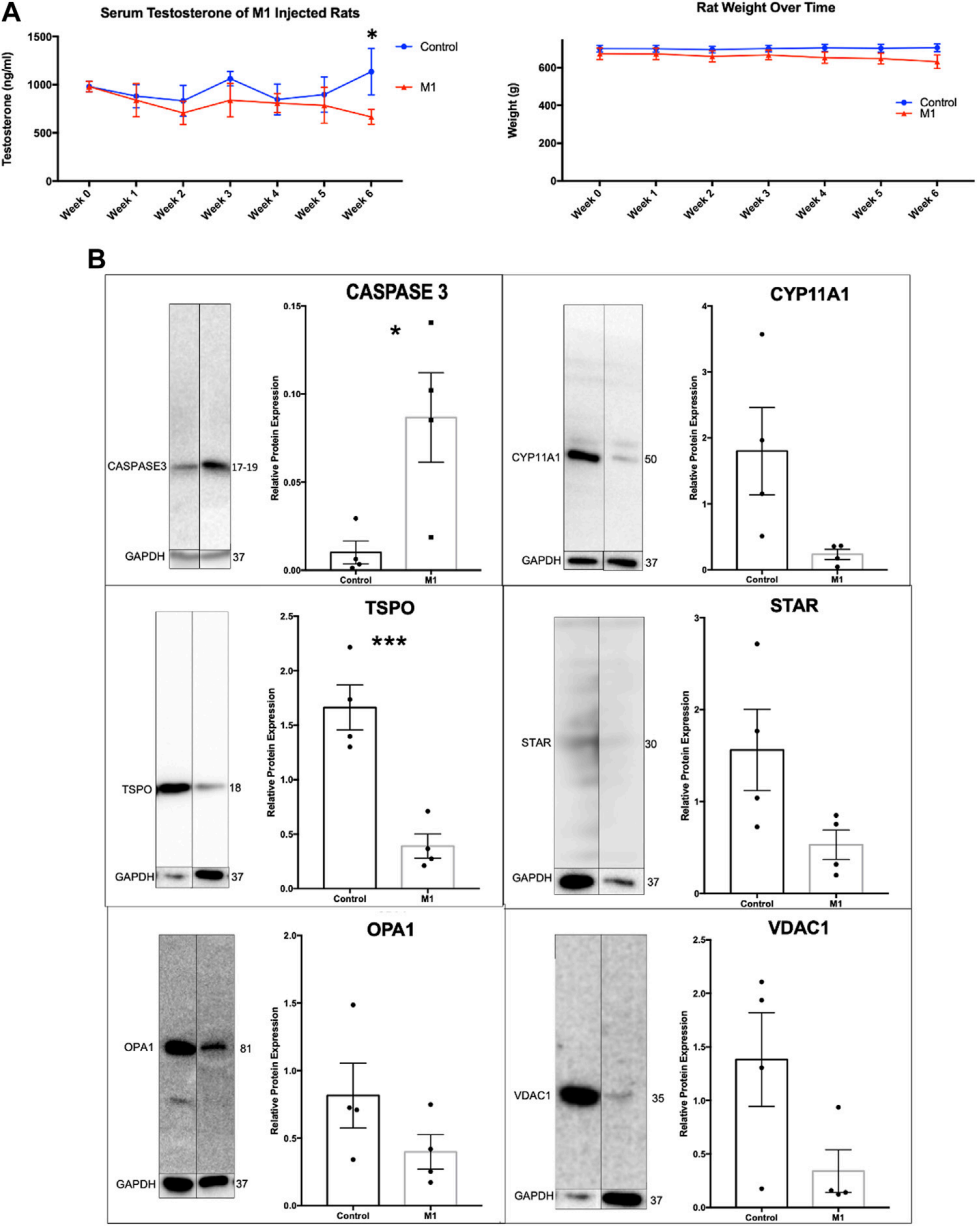
Administration of M1 leads to decreases in weight and testosterone levels in aged rats. **(A)** Rat weight and serum testosterone levels with M1 treatment over the experimental time course. **(B)** Representative immunoblots and comparative protein expression levels in Leydig cells from M1-treated and control aged rats. Data are presented as mean ± SEM. *p < .05, **p. < .01, and ***p < .001 by Student’s t-test.

Throughout the 6-week treatment period, serum and weight measurements were taken to assess the impact of a daily intraperitoneal injection of M1. M1 administered *in vivo* daily gradually decreased testosterone levels and weight over the 6-week treatment period when compared to untreated animals (Figure 3A), while isolated primary aged Leydig cells treated with M1 *ex vivo* had produced increased levels of testosterone (Figure 2A). Furthermore, analysis of primary Leydig cells isolated after the end of the *in vivo* treatment showed increased expression of apoptosis marker cleaved Caspase-3 and decreased expression of steroidogenic proteins (CYP11A1, TSPO, STAR, VDAC) (Figure 3B). Collectively, these data show declining Leydig cell health when M1 is administered *in vivo*.

### 3.4 Leydig cell mitochondrial integrity is compromised with M1-injected aged rats

**FIGURE 4:**
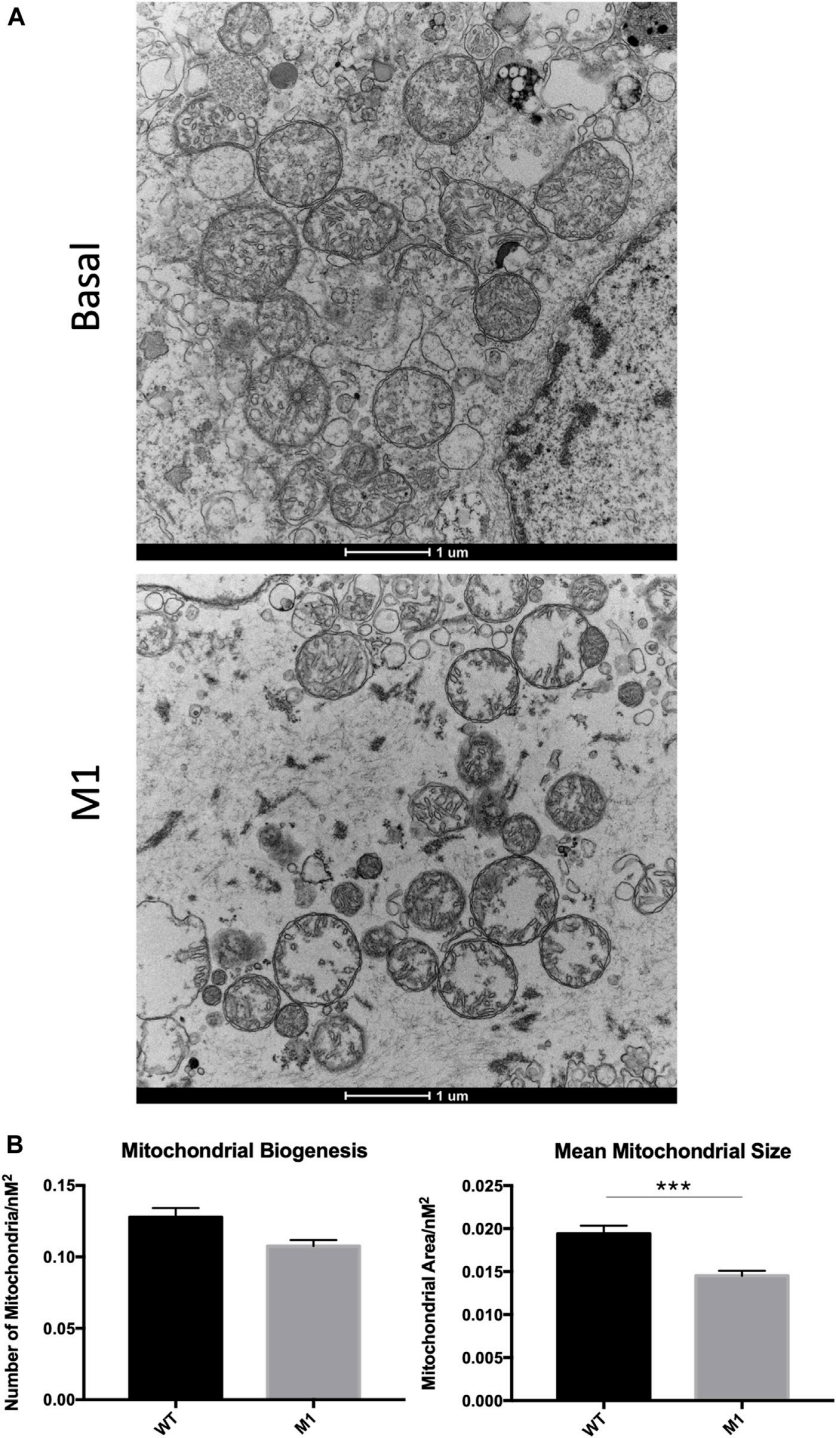
Leydig cell mitochondrial integrity is compromised in M1-treated aged rats. **(A)** TEM imaging of mitochondria from untreated basal and isolated primary Leydig cells from M1-treated aged rats. **(B)** Mitochondrial biogenesis and size of Leydig cell mitochondria. Data are presented as mean ± SEM. *p < .05, **p. < .01, and ***p < .001 by Student’s t-test. Scale bar, 1 μm. TEM, transmission electron microscopy.

Deterioration of mitochondria is associated with aging and observed in numerous diseases and conditions, so mitochondrial degradation was expected in aged Leydig cells here. Because the promotion of mitochondrial fusion enhanced mitochondrial phenotypes in previous studies, we were interested in visualizing the effects of M1 treatment on mitochondrial structure. After treatment was complete, Leydig cells were isolated, fixed, and observed using transmission electron microscopy, revealing abnormal phenotypic characteristics when compared with isolated cells from untreated animals (Figure 4A). Untreated Leydig cells displayed the expected normal degradation that occurs with aging. Weak mitochondrial integrity was observed in the untreated group, while mitochondria from treated animals displayed mitochondrial damage, including abnormal cristae structure and deterioration of the mitochondrial matrix. Additionally, the treated Leydig cells produced significantly decreased mitochondrial biogenesis and size (Figure 4B).

### 3.5 Effects of M1 treatment varies by tissue

**FIGURE 5:**
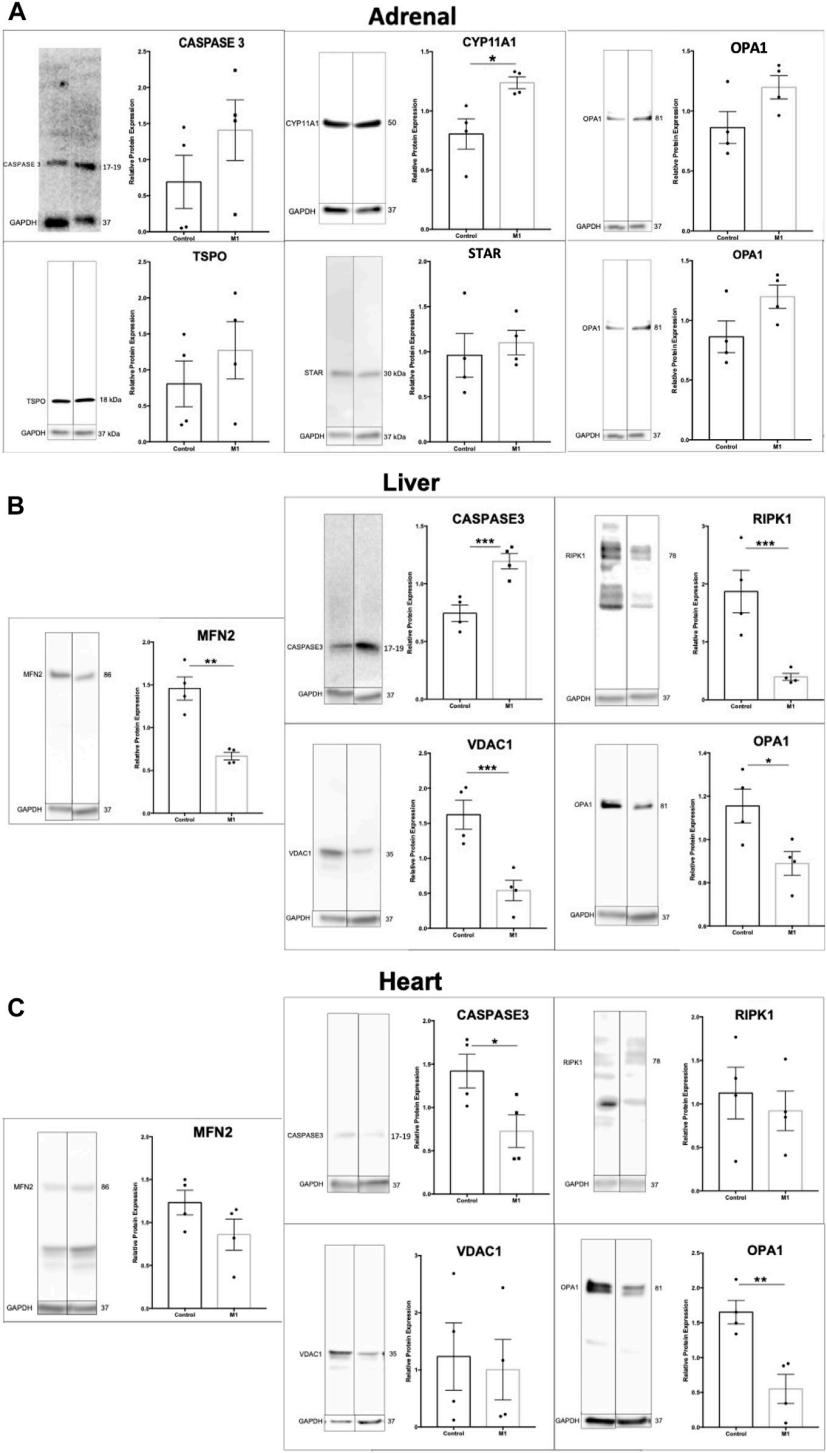
Effect of M1 treatment varies with tissue type. Representative immunoblot and protein expression values in adrenal **(A)**, liver **(B)**, and heart **(C)** samples from M1-treated aged rats. Data are presented as mean ± SEM. *p < .05, **p. < .01, and ***p < .001 by Student’s t-test.

Previous research using M1 have showed beneficial effects in a cardiovascular disease model and their healthy counterparts after injection with M1^33^. Using the same animals as above, adrenal, liver, and heart samples were evaluated. Immunoblots of extracted proteins from these tissues showed decreased markers for apoptosis and increased expression of some steroidogenic proteins (Figure 5A). Although the adrenal gland produces steroids, this production does not affect circulating testosterone levels. The data for adrenal cells suggest slightly increased health and increased steroidogenic capacity. Analysis of protein expression levels in liver samples showed significantly increased levels of apoptotic markers and decreased expression of mitochondrial proteins (Figure 5B), suggesting declining liver health and cell integrity after treatment. The expression of apoptotic markers decreased in heart samples (Figure 5C), confirming the beneficial effects found previously by Ding et al. (2020). Collectively, the effect of systemic M1 treatment assessed by protein expression varies at the tissue level, with some tissues showing beneficial health effects (adrenal, heart) and deleterious effects in others (testis, liver).

### 3.6 Prediction of ADME properties

**FIGURE 6:**
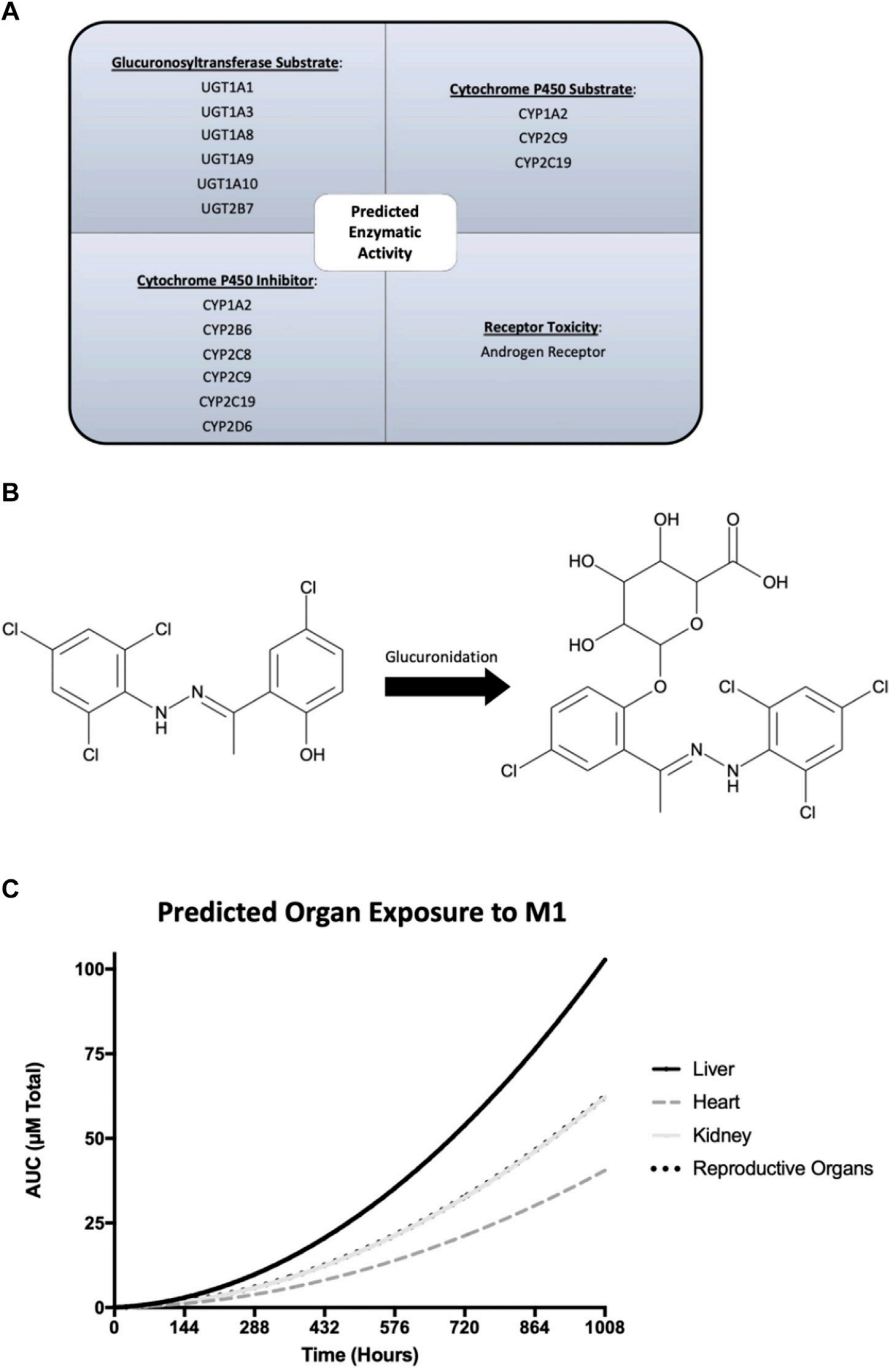
GastroPlus and ADMET Predictor modeling of the physicochemical, metabolism, and pharmacokinetic properties of M1. **(A)** Predicted enzymatic properties of M1 with glucuronosyltransferases and cytochrome P450 enzymes. **(B)** Molecular structure of M1 and its glucuronidated metabolite and its predicted exposure to tissues of interest over the course of the study. **(C)** Predicted plasma concentrations of M1 after injection over 6 weeks.

ADMET Predictor and GastroPlus software were used to predict ADME properties and simulate the *in vivo* rat experiment. Predicted physicochemical, metabolism, and pharmacokinetic properties of M1 were generated and used in physiologically based pharmacokinetic modeling. Quantitative structure activity relationship analysis through ADMET Predictor suggested that M1 is a substrate for numerous glucuronosyltransferases and cytochrome P450 enzymes (Figure 6A). Additionally, M1 was predicted to act as an inhibitor of several cytochrome P450 enzymes and could display androgen receptor-related toxicity. Next, plasma concentrations were predicted using the actual experimental conditions for dose and route of administration as input settings in the pharmacokinetic model. The results suggest that there should be no accumulation of M1 after multiple dosing and it should be cleared prior to subsequent doses. To better understand the varying effects amongst tissues that were observed, as shown in Figure 4, a physiologically based pharmacokinetic model of a rat was developed to estimate specific organ exposure to M1. Total exposure to the native M1 compound was highest in the liver, followed by the reproductive organs and kidney. Furthermore, since M1 is highly hydrophobic it was predicted to undergo glucuronidation as a possible phase 2 metabolic clearance mechanism to facilitate excretion (Figure 6B).

## 4 Discussion

Age-related Leydig cell decline has been associated with multiple cellular changes, including reduced expression of steroidogenic enzymes (Zirkin and Chen, 2000), increased antioxidant levels (Sokanovic et al., 2021), and declining mitochondrial function (Papadopoulos and Zirkin, 2021; Garza et al., 2022). The development of therapeutics that ameliorate these characteristics would greatly advance our ability to address the underlying issues contributing to age-related testosterone decline. Mitochondrial dynamics play a significant role in regulating cellular function but declines with aging (Zirkin and Chen, 2000; Midzak et al., 2009; Zacharioudakis and Gavathiotis, 2023). Interestingly, the expression of key steroidogenic proteins has been associated with changes to mitochondrial dynamic activity, after altering mitochondrial membrane fusion led to changes in the expression of SITE proteins (Duarte et al., 2012; Garza et al., 2022).

When MACS-isolated aged Leydig cells were treated with M1 *ex vivo*, a mitochondrial fusion promoter, the results showed beneficial effects on cell health and steroidogenic output, suggesting that Leydig cell health and function may be regulated by modulating mitochondrial dynamics. After treatment, these aged Leydig cells showed higher levels of mitochondrial bioenergetics and testosterone formation. Improved oxygen consumption indicated that the mitochondria in treated Leydig cells were more active and able to meet increased cellular energy demands and, in addition, had an increased capacity for steroid hormone formation. These findings are in agreement with our earlier preliminary study where M1 partially recovered testosterone formation in isolated Leydig cells from aged rats *in vitro* but it did not affect androgen formation in Leydig cell isolated from young rats (Garza et al., 2022) The present findings highlight a strong relationship between mitochondrial dynamics and Leydig cell function and suggest that carefully altering mitochondrial fusion may produce improvements to Leydig cell health, function, and longevity in aging models. However, these findings were not confirmed *in vivo*. Rats treated with M1 for 6 weeks produced slightly lower levels of testosterone and lost weight over the course of the study, suggesting that the treatment was detrimental.

The discrepancy between these two models, the beneficial effects seen in the isolated cell model and contrasting results in the 6-week injection treatment, is insightful, nonetheless. *Ex vivo* treatment promoted mitochondrial membrane fusion and led to mitochondrial biogenesis, improved mitochondrial function, and enhanced steroid hormone output. Overall, the health and integrity of Leydig cells increased when treated *ex vivo*. There could be several reasons for this discrepancy. One possibility is that systemic administration may have had unintended off-target effects in our animal model that may have altered or disrupted other physiological functions. This was demonstrated when we looked at other tissues and found either beneficial or deleterious effects depending on tissue type. In addition, long-term exposure to the compound may produce negative side effects; a possibility that may not have been observed in short-term studies (Garza et al., 2022). By contrast, the use of mitochondrial fusion promoters intermittently may promote mitochondrial health by temporarily enhancing the mitochondrial network (Zacharioudakis and Gavathiotis, 2023). Previous studies showed that healthy MA-10 Leydig cells may suffer from hyperfusion of mitochondria after M1 treatment (Garza et al., 2022), though we did not see that in our isolated primary Leydig cells. Exploring the effects of long-term and intermittent exposure to M1 in the MA-10 Leydig cells would be valuable.

It is worth noting that other pharmacological and metabolic properties play a role in drug exposure. Differences in pharmacokinetic and pharmacodynamic properties could significantly alter course of a compound *in vivo.* Therefore, thorough dosage and PK/PD studies are needed to understand the mechanisms involved. At present, we do not know how M1 is metabolized, or whether toxic metabolites are generated from its metabolism. M1 is a hydrophilic compound known to promote mitochondrial fusion^42^. Upon intraperitoneal administration, it predominately gets absorbed through the portal vein and is subject to initial hepatic first pass metabolism. Quantitative structure activity relationship analysis using ADMET Predictor software indicated that the liver should robustly metabolize M1, given its phenolic structural, which acts as a potential substrate for multiple glucuronosyltransferases. As illustrated in Figure 6, this characteristic may result in differential drug exposure across organs. Notably, while M1 exhibited detrimental impacts on the liver and testis, it conferred benefits to the heart. Our physiologically based pharmacokinetic model of the rat revealed that the heart probably experienced the least exposure to M1 throughout the study. The simulated rat model suggests that lower systemic doses of M1 may yield positive outcomes and that M1’s predicted pronounced hepatic metabolism could be linked to the specific route of administration and the observed detrimental effects in liver.

Mitochondrial fusion has emerged as a promising therapeutic strategy and target for improving cell health and function (Garza et al., 2022; Zacharioudakis and Gavathiotis, 2023). Mitochondrial dysfunction is a hallmark of numerous pathologies (Sisková et al., 2010; de La Barca et al., 2016), and implementing strategies which could regulate, and control mitochondrial health would significantly improve our ability to treat pathologies related to metabolic dysfunction. Numerous studies have confirmed that mitochondrial dynamics are essential for the proper growth, maintenance, and specialization of stem cells, as well as for the overall health of other cells (Zacharioudakis et al., 2022). Moreover, it has been demonstrated that correcting imbalances in mitochondrial dynamics can slow or even prevent the development of various diseases (Rocha et al., 2018; Ferreira et al., 2019; Franco et al., 2020). The proteins that regulate mitochondrial dynamics are becoming increasingly recognized as a new group of potential therapeutic targets (Zacharioudakis and Gavathiotis, 2023). Promoting mitochondrial fusion may be one approach to enhancing cell health and wellbeing with aging, but more investigations are warranted. Our findings suggest that fusion promoters could potentially enhance the productivity of aged Leydig cells when carefully regulated.

## Data Availability

The original contributions presented in the study are included in the article/Supplementary material, further inquiries can be directed to the corresponding author.
